# Spatiotemporal Omics-Refining the landscape of precision medicine

**DOI:** 10.1093/lifemedi/lnac053

**Published:** 2022-11-14

**Authors:** Jiajun Zhang, Jianhua Yin, Yang Heng, Ken Xie, Ao Chen, Ido Amit, Xiu-wu Bian, Xun Xu

**Affiliations:** BGI Research-Southwest, BGI, Chongqing 401329, China; JFL-BGI STOmics Center, Jinfeng Laboratory, Chongqing 401329, China; BGI Research-Shenzhen, BGI, Shenzhen 518083, China; BGI Research-Shenzhen, BGI, Shenzhen 518083, China; BGI Research-Shenzhen, BGI, Shenzhen 518083, China; Department of Immunology, Weizmann Institute of Science, Rehovot, Israel; BGI Research-Southwest, BGI, Chongqing 401329, China; JFL-BGI STOmics Center, Jinfeng Laboratory, Chongqing 401329, China; BGI Research-Shenzhen, BGI, Shenzhen 518083, China; Department of Immunology, Weizmann Institute of Science, Rehovot, Israel; Department of Pathology, the First Affiliated Hospital of University of Science & Technology of China, Hefei 230036, China; Chongqing Institute of Advanced Pathology, Jinfeng Laboratory, Chongqing 400038, China; BGI Research-Southwest, BGI, Chongqing 401329, China; JFL-BGI STOmics Center, Jinfeng Laboratory, Chongqing 401329, China; BGI Research-Shenzhen, BGI, Shenzhen 518083, China

**Keywords:** spatiotemporal omics, precision medicine, pathomechanism of disease, spatial algorithms, technologies of spatial omics

## Abstract

Current streamline of precision medicine uses histomorphological and molecular information to indicate individual phenotypes and genotypes to achieve optimal outcome of treatment. The knowledge of detected mutations and alteration can hardly describe molecular interaction and biological process which can finally be manifested as a disease. With molecular diagnosis revising the modalities of disease, there is a trend in precision medicine to apply multiomic and multidimensional information to decode tumors, regarding heterogeneity, pathogenesis, prognosis, etc. Emerging state-of-art spatiotemporal omics provides a novel vision for in discovering clinicopathogenesis associated findings, some of which show a promising potential to be translated to facilitate clinical practice. Here, we summarize the available spatiotemporal omic technologies and algorithms, highlight the novel scientific findings and explore potential applications in the clinical scenario. Spatiotemporal omics present the ability to provide impetus to rewrite clinical pathology and to answer outstanding clinical questions. This review emphasizes the novel vision of spatiotemporal omics to refine the landscape of precision medicine in the clinic.

## Introduction

Emerging new treatment modalities like immune checkpoint inhibitory therapy not only offers impetus to facilitate precision medicine of cancer but also raises the urgent build-up of a new system to guide disease management [1]. Pathology is the conclusive diagnostic tool to set the rational basis for clinical care and therapy [2]. Regarding cancer, pathology is the gold standard to guide treatment and prognosis. However, complex diseases like cancer can hardly be defined as one disease with its multifactorial nature. The heterogeneity of cancer requires multiple dimensions of information to decode the etiopathogenesis of cancer [3]. Current clinic-accessible assays hinder the possibilities of revealing the nature of clinical manifestation, courses, and outcomes.

In recent decades, the development of biotechnologies has provided different perspectives on understanding diseases. However, a limited number of novel biotechnologies have proof of evidence for the clinicopathologic correlation. Alteration in cellular and tissue organization detected by histopathology lays the foundation of pathology [4], which emphasizes the significance of spatial organization in pathology. Morphological features distinguish benign from malignant tumors, and define stages and grading. Immunohistochemistry (IHC) assays assist in characterizing undifferentiated malignant tumors, the origin of metastatic tumors, and providing some molecular evidence for prognosis and treatment decision [4]. In situ hybridization and IHC are universal tools in the clinic to offer diagnosis evidence of molecular expression with geographic location in tissue [4]. There is a trend in precision medicine to apply comprehensive genome information to decoding tumors, some of which have been translated into clinical diagnostics to guide treatment decisions. Massive gene mutation data can be one part of the puzzle to explain how a tumor is manifested to impact the biological functions and finally, a person’s health. However, the evident limitation of these assays is that they are missing critical information which can lead towards much more precise decisions regarding optimal treatment of the patients.

**Figure 1.**
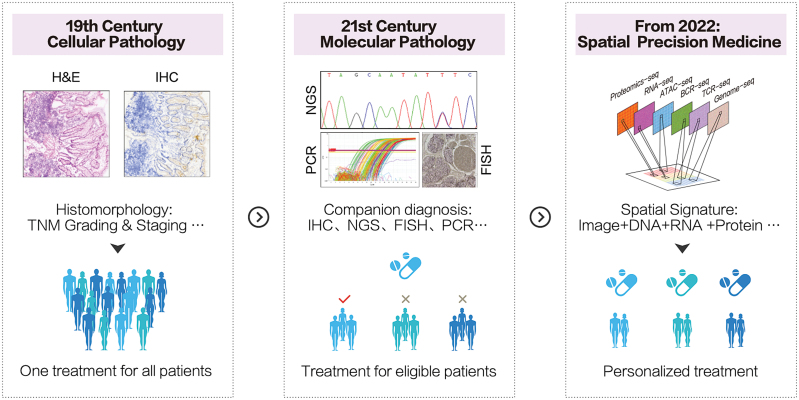
**History and future of precision medicine.** Development of featured techniques in each period facilitates advancement of precision medicine. Histomorphology-based modalities have been the core of diagnosis by providing evidence of alteration in architecture and function. Molecular assays can stratify patients eligible for targeted therapy. The emerging of spatiotemporal omic techniques represents a new era of precision medicine.

Given the obvious limitations of clinic assessable technologies, spatiotemporal omics possess the ability to define the relevant target cells and molecules harboring pathologically related signatures by high-throughput data [5]. Numerous researches report comprehensive cellular communication, complex regulatory networks, cancerous heterogeneity, and homeostasis of microenvironment with spatiotemporal omic technologies. Heterogeneity of associations within and across histomorphological types can be indicated and unmasked by spatial relations with visualization of profile [6]. Computational algorithm enables reconstruction of two-dimensional mapping into a 3D atlas, which facilitates the investigation of heterogeneity on a scale of organoids that can hardly be represented by analyses of sections [7]. Other than diagnosis based on alteration in architecture and function [2], spatiotemporal omics guarantee new modalities of molecular subgrouping of diseases referring to underlying pathomechanism, which avoids diagnostic inconsistencies and therapeutic resistance. Even though the quantification of clinical aggressiveness of tumors is proven to be highly associated with prognosis, it is difficult to be detected accurately with current technologies. With spatial information, architecture, and organization like tertiary lymphoid structures and invasive leading edges show the critical roles in tumor progression and antitumor response. In addition, molecular signature has been observed in domain feature. Spatial signature composing of selected molecules and coordination on tissue have better performance of accuracy and sensitivity than traditional stratification method in predicting treatment response and prognosis. Therefore, the spatiotemporal omic technologies promise great potential of promoting clinical practice with sensitive biomarker system, accurate patient stratification, and precise prediction of risk (summarized in Fig. 1).

Here, we summarize available spatiotemporal omics technologies and highlights scenario of application in facilitating discoveries in the etiopathogenesis of cancer. By reviewing mathematical algorithms and computational tools, this paper will support initial principles of clinical and spatial data integration. In addition, from the inception of research frontier, this review emphasizes the promise of spatiotemporal omics to mark the beginning of a revolution in precision medicine and indicates the unresolved clinical and computational challenges.

## Technologies of spatial omics: all about what and where

In recent years, a growing number of spatial transcriptomic (ST) technologies have been developed to enable gene expression profiling of tissues with spatial information such as GEO-seq [8], MERFISH [9], ST [10], *in situ* sequencing (ISS) [11], etc. And these ST profiling methods have been extensively reviewed elsewhere [6, 12, 13]. However, transcriptomic alone can hardly distinguish immune cell subtypes with similar transcriptic profiles in single-cell sequencing [14]. Thus, it is necessary to develop spatial multiomics methods which could thoroughly describe physiological and pathological process with integrated information on omics and architecture. In this section, we mainly focus on the methods that have been developed to enable spatial analysis of RNAs and proteins on the same tissue section. Those technologies can be roughly divided into three categories based on how these methods acquire spatial information: imaging-based, sequencing-based, and microdissection-based methods.

### Imaging-based methods for spatial omics

In imaging-based methods for co-detection of RNA and protein, RNA signals are usually generated from probes designed to bind *in-situ* mRNA targets, and protein signals are detected via specific antibodies. Different labeling strategies could be used to distinguish different probes and antibodies, like isotope-conjugation or DNA-barcoding. Depending on labeling strategies, different imaging systems are utilized to visualize spatial location of the targeted RNAs and proteins.

Co-Detection by Indexing (CODEX) is a highly multiplexed protein *in situ* detection platform, which is based on sequential cycles of fluorophore-labeled probe hybridization and imaging [15]. Unique DNA oligos are conjugated to different antibodies, thus enabling multiplexed protein detection. The PhenoCycler^TM^-Fusion system (commericalized CODEX platform by Akoya Biosciences) can detect 100+ proteins at subcellular resolution (200 nm) with quite large imaging area (18 mm × 34 mm) [16]. Nevertheless, co-detection of RNA and protein is not possible in the current workflow. To this end, Cheng *et al.* combined CODEX and RNAscope, enabling simultaneous visualization of RNA and proteins on the same section [17]. Recently, RNA and protein co-detection workflow has been established on PhenoCycler^TM^-Fusion system, which enables 102-plex RNA and 50-plex protein co-detection on the same tissue section. However, this solution is not commercially available yet. Although spatial omics profiling can be achieved on the CODEX platform, however, there are several limitations: (i) Because of the spectral overlap, multiple rounds of hybridization, imaging, and stripping steps are required to realize multiplexed protein detection [15]. (ii) the platform currently lacks a signal amplification step, which might be an issue for detection of low-abundant protein. To this end, methods with signal amplification have been developed, such as Immuno-SABER and InSituPlex.

CosMx^TM^ Spatial Molecular Imager (SMI) from NanoString also uses sequential cycles of probe hybridization and imaging strategy for both RNAs and proteins detection. This imager could detect up to 1000-plex RNA or 100-plex protein at subcellular resolution (200 nm), and with high sensitivity (1–2 copies/cell), low background (~0.04 counts/cell) and low error rate (0.0093 false calls/cell) [18]. However, current solution from SMI does not support RNA and protein co-detection. Further improvements on chemistry and workflow are needed.

Instead of using DNA oligos as an antibody identifier in CODEX, unique rare earth metal isotopes can also be used to label probes and/or antibodies. UV or ion beam is next applied to ablate tissue spot by spot [19, 20]. The ablated materials are further analyzed by mass spectrometry-based imaging system to identify probe and/or antibody types. Using isotope-labeled probes and isotope-conjugated antibodies, Schulz *et al.* showed that multiplexed imaging of mRNA and proteins on the same tissue section can be achieved via mass spectrometry [21]. Mass spectrometry-based imaging systems include imaging mass cytometry and multiplexed ion beam imaging by time-of-flight (MIBI-TOF). The MIBI-TOF can now reach quite high-spatial resolution (down to 260 nm) [20]. In addition, mass spectrometry could detect all the metal isotopes simultaneously without interference (no autofluorescence issue), thus these methods do not require multiple rounds of imaging [22]. However, the relatively small fields of view (up to 1 mm^2^), limited multiplexibility (up to 40-plex for MIBI-TOF now) and difficulties in accessing mass spectrometry limitate its broad application [20].

### Sequencing-based methods for spatial omics

In sequencing-based methods for spatial omics, one common approach is to use barcode probes on microarray to capture mRNAs and antibody-oligonucleotide conjugates. Next, the spatial information is introduced by *in situ* reverse transcription reaction. After cDNA release and library preparation, gene and protein expression profiles are subsequently quantified using next-generation sequencing (NGS).

ST (or Visium) uses DNA oligo with known spatial coordinates directly linked to the glass slide [10]. For Stereo-seq, DNA nanoballs (DNBs) with unknown spatial coordinate sequences are first loaded onto a two-dimensional space, which is followed by sequencing to profile the spatial coordinates for each DNB. Then, capture probes are further generated based on the spatially profiled DNB [23]. In the end, both Visium and Stereo-seq generate capture probes with known spatial coordinates for capturing multiomics information. Several RNA and protein co-detection workflow have been developed on ST/Visium platform [24, 25]. 10× Genomics also has developed RNA and protein co-detection workflow [26]. But the Visium FFPE V2 kit for spatial gene and protein expression profiling in FFPE tissues has not been commercially available yet. All above-mentioned workflows/solutions can achieve whole transcriptome and multiplexed protein detection. The maximum protein multiplexity reported now is 32-plex [25]. BGI is also developing its own RNA and protein co-detection workflow based on Stereo-seq. Due to its unique DNBSEQ technology, Stereo-seq can reach a much higher resolution with 0.5 µm spot center-to-center distance as compared to ~100 µm in Visium [23]. Details with high resolution are expected to be obtained through this workflow. However, current solid-phase capture methods such as Visium and Stereo-seq both have dead-space areas in their patterned arrays, where no information is captured. In addition, lateral molecule diffusion after permeabilization is another limitation [13].

Different from solid-phase capture methods like Visium and Stereo-seq, deterministic barcoding in tissue for spatial omics sequencing (DBiT-seq) is based on a two-step microfluidic-delivery of DNA barcoded oligo to the tissue surface to capture mRNA or antibody-derived tag information [27]. The latest spatial-CITE-seq can co-detect whole transcriptome and up to 273 proteins [28]. In addition, DBiT-seq has been further developed to combine spatial ATAC-seq [29] and CUT&Tag [30]. Although higher capture efficiency is observed compared to solid-phase based method such as ST and Slide-seq [27], there are several limitations due to the usage of the microfluidic system: (i) Dead-space areas are inevitable. (ii) There is the theoretical limit of pixel size. The highest resolution so far is 10 µm [27], which means it is not single-cell level resolution in the strict sense. (iii) There is certain degree of diffusion between microchannels. (iv) Tissue deformation exists.

For Visium, Stereo-seq and DBiT-seq, *in situ* generated cDNA is released from the tissue section, and subsequent library preparation and sequencing are performed *ex situ*. Another approach is to directly perform sequencing on the tissue, namely ISS. ISS-based transcriptomic profiling methods such as FISSEQ, BaristaSeq, STARmap, and INSTA-seq have been extensively reviewed elsewhere [6]. The advantage of ISS-based approaches is that there is no dead-space area, and with higher spatial resolution compared to Visium, Stereo-seq, and DBiT-seq. However, there are some limitations of ISS-based methods: (i) Unlike above mentioned sequencing-based methods, ISS can not perform whole transcriptome analysis yet. (ii) No multiomics workflow has been established yet. But it is theoretically possible at least to incorporate protein detection into the current workflow, as antibody-derived DNA tags also can be sequenced by ISS. At the end of 2022, 10× genomics is planning to launch the Xenium ISS platform with multiplexing capability up to ~400 genes. However, protein detection will not be possible on the first version of this platform.

### Microdissection-based methods for spatial omics

Unlike sequencing-based and imaging-based methods, microdissection-based methods can only perform multiomics analysis on the selected region of interest (ROI) instead of profiling the whole landscape of the tissue. One common technique to isolate ROI is laser capture microdissection (LCM). So far, LCM has been widely used in coupled with RNA sequencing [31], DNA mutation detection [32] and proteomics study [33]. Similarly, via directly microbiopsy punching on the cryosectioning, sciMAP-ATAC is able to obtain a spatially resolved epigenetic profile [34]. Another special example is the GeoMx Digital Spatial Profiler (DSP) from NanoString, which combines ROI selection and sequencing together. For DSP workflow, users need to first stain the tissue section with imaging and profiling reagent. Next, ROIs are selected and UV light is applied to the selected area to release unique oligos from profiling reagents. The released oligos are hybridized to NanoString® barcodes and quantitated by the nCounter platform (or NGS) [35]. For now, DSP could simultaneously detect up to 1000 proteins or whole transcriptome (only in human and mouse now) [36, 37]. However, the current workflow does not support RNA and protein co-detection on the same tissue section. NanoString already has released GeoMx spatial proteogenomics workflow for RNA and protein co-detection. But this solution is not commercially available yet. The main limitations of these microdissection-based methods are the relatively low resolution and limited number of ROIs could be isolated.

Current imaging-based spatial omics methods provide subcellular resolution but with relatively low-throughputs. Whereas sequencing-based methods such as ST and DBiT-seq provide high-throughputs but with relatively low-spatial resolution. Few sequencing-based methods such as Stereo-seq, Seq-Scope [38], and PIXEL-seq [39] can generate ST data with sub-micron resolution, but spatial omics workflow is still lacking. Thus, new spatial omics technologies with high-spatial resolution and high-throughput are still needed to be developed. In addition, giving spatial omics temporal dimension is also of vital importance especially for investigation of development and disease progression. Some spatially resolved developmental trajectories of mouse organogenesis [23], *Drosophila* embryogenesis [7], zebrafish embryogenesis [40], chicken cardiogenesis [41], and liver regeneration after acute injury [42] have been constructed using Stereo-seq or Visium based on transcriptome information. In the future, adding proteomic, metabolomic, epigenetic, and even 3D configuration to the current transcriptomic data would enable researchers to gain more comprehensive and valuable insights into the biological process. At that time, spatiotemporal omic technologies for sure will be a valuable tool for understanding the development and progression of complex disease and designing potential therapies.

## Algorithms for the analysis of spatially resolved data

As summarized in last section, multiple technologies have been available to detect spatiotemporal omic information. However, the main challenges are to realize proper handling and interpretation of massive data, and a unified standard of combining multidimensional data. The goal of precision medicine is to personalize therapy for each individual. Achieving this goal requires integrating massive amounts of personalized data, including omics data, clinical imaging, biochemical test data, medical history, mental condition, social, and environmental determinants. Diseases such as tumors are highly heterogeneous, leading to immune responses, and outcomes after treatment varying across different patients. It is best to examine the pathological tissue at the molecular level with spatially accurate information to understand the biomarker distribution, and monitor the development and prognosis of the disease. Therefore, recently developed spatiotemporal omics technology is a natural complement to the current data integration of mechanistic research and precision medicine.

Specifically, spatiotemporal omics can:

1. provide spatial clustering information to illustrate tissue structures;
2. perform cell annotation to study the spatial composition of tissues;
3. analyze gene co-expression to exhibit biomarker distribution;
4. detect cell–cell interaction to guide medical treatment and drug discovery.

**Figure 2.**
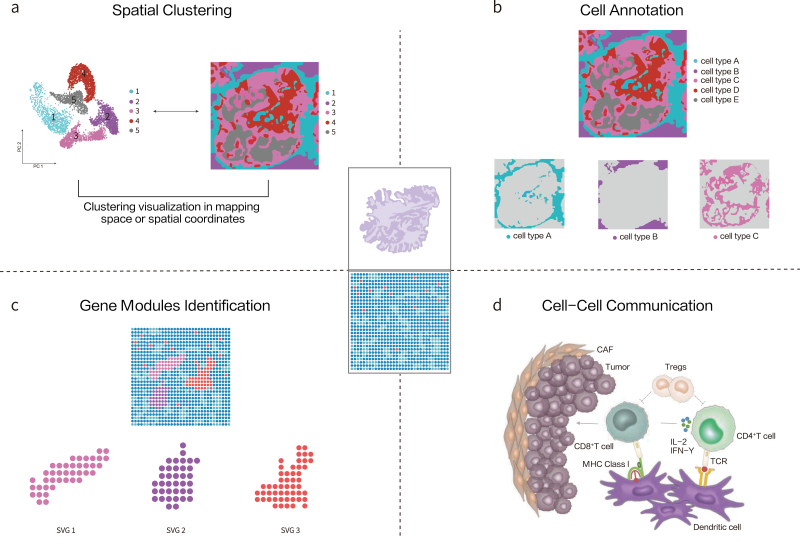
**Algorithm applications in pathological analysis.** (a) Illustrate tissue structures by spatial clustering. Clustering result can be visualized in the mapping space or spatial coordinates. (b) Study the spatial composition of tissues by performing cell annotation. Each cell type can be studied separately. (c) Exhibit biomarkers (spatial variable genes) distribution by gene modules identification. Each biomarker has different expression pattern. SVG, spatial variable gene. (d) Guide medical treatment and drug discovery by detecting cell–cell communication. The communications between tumor cells and immune cells in the diagram are as an example.

The following sections briefly introduce a few selected algorithms in ST, together with their applications in pathological analysis to get a glimpse of the potential of advancing understanding in pathological mechanism, biomarker identification, and drug discovery. It is not meant to be a comprehensive review of analytical methods but an explanation of what ST analysis can provide to broaden the horizon of precision medicine. The included methods are summarized in Table 1 and Fig. 2.

**Table 1.** Summary of omics data integration tool

Methods	Analysis	Description	URL
BayesSpace	Spatial Clustering	A Bayesian statistical model for clustering analysis and resolution enhancement.	https://github.com/edward130603/BayesSpace
SEDR		A deep autoencoder to embed gene expression and spatial information for clustering.	https://github.com/JinmiaoChenLab/SEDR
SpaGCN		A graph convolutional network for gene expression, spatial location and histology image integration.	https://github.com/jianhuupenn/SpaGCN
RCTD	Cell Annotation	A supervised learning model to decompose cell types.	https://github.com/dmcable/spacexr
Cell2location		A Bayesian model to map cell types spatially.	https://github.com/BayraktarLab/cell2location
CoSTA	Gene Module	A convolutional network based feature extraction method that is capable of detecting correlated spatial gene patterns.	https://github.com/rpmccordlab/CoSTA
Hotspot		A gene module detection method based on graph-based similarity measures.	https://github.com/YosefLab/Hotspot
DAGBagST	Cell–cell Interaction	A statistical model combining spatial and molecular information to construct ligand-receptor networks.	Currently unavailable
CellphoneDB		A ligand-receptor database and a protocol for cell–cell communication analysis.	https://github.com/ventolab/CellphoneDB

### Spatial clustering

Spatial clustering is the basic yet vital step in ST analysis. It partitions tissue regions according to biological patterns, providing a global view of the tissue structures and functions. Zhao *et al*. [43] developed BayesSpace, a fully Bayesian model with a Markov random field prior to perform spatial clustering. The top principal components were extracted as input after principal component analysis to top highly variable genes. The model parameters were updated iteratively and sequentially by Gibbs sampling and Metropolis-Hastings, which were both Markov chain Monte Carlo methods. BayesSpace was applied to squamous cell carcinoma data, and the delineated borders between different major cell types or regions matched the pathologist’s annotations. Specifically, the basal and suprabasal keratinocytes showed spatially distinct expression, while the melanocytes were concentrated around the hair follicle, and the myeloid and T cells gathered in the stroma and immune infiltration regions. Fu *et al*. [44] introduced SEDR to embed gene expression with spatial location. A deep autoencoder was used to obtain a low-dimensional feature space from gene expression, and a variational graph autoencoder was concurrently applied to integrate gene expression with spatial information to produce a spatial embedding. Then an iterative clustering method was employed to the concatenated embeddings to output the spatial clustering result. For the 10× Visium ST data of human breast cancer, SEDR produced many subclusters within the tumor region, exhibiting the capability of delineating tumor and nontumor regions, and assessing intratumoral heterogeneity. SpaGCN [45] constructed an undirected weighted graph by incorporating the gene expression and corresponding histology information, where the edges between every two nodes were determined by the spatial Euclidean distance. The integrated features were aggregated by a graph convolutional network, and the spatial clusters were extracted by an unsupervised iterative clustering algorithm. The authors verified the algorithm on a human primary pancreatic cancer dataset, and further showed that with additional histological information, the spatial clusters corresponded well to the tumor regions, and the identified marker genes KRT17 and MMP11 matched the prior knowledge of the pancreatic cancer. Spatial clustering exhibits global information of tissue composition, and lays a foundation for the following detailed molecular analysis. The various hierarchical structures of different patients urged a personalized analysis of the pathological mechanism, which could eventually lead to customized medical treatment.

### Cell annotation

Spatial clustering illustrates the tissue structures on a broader scale, while cell annotation takes our understanding to the molecular level. Biologically, different cell types or cells at different states display distinct gene expression levels, and those with similar expressions are assigned the same cell identity. According to the gene expression of each cell, Cable *et al*. developed Robust Cell Type Decomposition (RCTD) for decomposing cell type mixtures in ST [46]. The annotated scRNA-seq data was used to define the cell type expression profile, and a statistical model was trained to estimate the pixel-wise cell type mixtures. Taghreed Hirz *et al*. annotated cell identities within the prostate tumor microenvironment (TME) with RCTD on both 10× scRNA-seq data and Slide-seq data [47]. They first grouped and annotated cells into major clusters: T cells, B cells, stromal cells, epithelial cells, and myeloid cells. Then each cluster was extracted for finer subclusters annotation. Cell2location is a Bayesian model for ST cell type mapping [48]. The algorithm first estimated the average gene signatures of each identified cell cluster with Negative Binomial regression. Then the reference cell types from scRNA-seq data of the same tissue type were used to decompose and estimate the cell identities at each spatial location. The authors applied Cell2location to the human lymph node and disentangled a rare pregerminal center B-cell population from the interlaced immune cells. By observing a distinct gene signature that suggests interferon response, they predicted a putative cellular interaction corresponding to viral infection. Lopez *et al*. proposed DestVI [49], a probabilistic method with two autoencoders to jointly model the reference scRNA-seq data and the ST data and deconvolute the cell type proportions for every measuring spot as well as the average cell states for each cell type simultaneously. The authors tested DestVI on the murine lymph nodes and mouse tumor model to show the effectiveness of cell type deconvolution in analyzing the spatial organization of various types of immune cells within the microenvironment. Except for metrics based on deconvolution, many researchers have made numerous efforts to annotate ST at single-cell-level. Tangram align sc/snRNA-seq data in space to match “the shape” of the spatial data as “puzzle pieces”, by maximizing the correlation between the gene profiling of spatial and single-cell transcriptomics from random [50]. La Manno utilized Tangram to align single-cell transcriptomics to ST in mouse brain [51]. Besides annotating cell types, Tangram can also impute spatial expression patterns of genes which are not directly detected. In addition, Wei *et al*. developed Celltrek [52], a method which projects single cells to their spatial coordinates in tissue sections by embedding ST and scRNA transcriptomics to a shared feature space. By applying Celltrek to DCIS (ductal carcinoma in situ) sample, the author found that the subclones of tumor with different copy number alterations had spatial heterogeneity. Moreover, CellDART was proposed to infer cell types by adversarial discriminative domain adaptation [53] and has been used to identify cell types and molecular markers of a mouse xenograft tumor with nanomedicine distribution [54]. Cell annotation opens the door to exploring the tissue microenvironment at the molecular scale, providing a deeper understanding of human organ developments, immune system response, as well as prognostication after treatment, which facilitates studies of biomarker identification and cellular engineering for precision medicine.

### Gene module analysis

A gene module is a group of genes that are spatially similar or have similar expression levels. The co-expression gene modules provide information to study the spatial pattern and their potential regulatory behaviors, and eventually gain knowledge on the functions and significance of specific genes. Xu and McCord presented CoSTA to learn a spatial relationship of gene expressions [55]. The expression matrix of each gene in ST data was concatenated and used as input to a 3-layer convolutional network for feature extraction. A Gaussian mixture model was applied to the network output to generate a soft clustering, which was used as a label to train the network. The author tested the method on brain-injured mouse samples and discovered that the spatial gene patterns of *Vim, Ctsd,* and *Gfap* were more variable in the acute phase than in the samples 2 weeks after injury. DeTomaso and Yosef introduced a general method called Hotspot to detect informative gene modules [56]. It utilized a spatially informed similarity graph to extract features that represented the highly variable gene expression patterns and then clustered the pairwise correlation to form meaningful gene modules. Taghreed Hirz *et al*. [47] applied Hotspot to calculate the spatial autocorrelation of tumor and nontumor regions. This provided a quantitative evaluation of the dispersed organization of fibroblasts, pericytes, endothelial cells, and others in the tumor regions, in contrast to the well-organized structures in healthy or adjacent-normal tissues, and helped characterize the TME of prostate cancer. Yang *et al*. [57] found that the tumor progression resulted in cell clusters with different transcriptional states, which coincided with the three transcriptional gene modules detected by Hotspot. The expression of the gene modules could be predictive factors for the survival rate of lung adenocarcinoma patients. The gene module analysis helps to visualize the expression distribution within the tissue structure and indicates potential biomarkers for diagnosis, treatment, and prognosis.

### Cell–cell interaction

The human body is a complex ecosystem composed of different types of cells, which interact with one another with specific biochemical signaling. It is essential to understand intercellular communication to explain the functional changes in diseases relative to healthy conditions. Chowdhury *et al*. developed DAGBagST to characterize cell–cell interaction utilizing spatial context in ST data [58]. The gene expression and spatial information were integrated to build neighbor integrated matrices. Then a directed acyclic graph was used to construct the ligand-receptor network. DAGBagST was applied on high-grade serous ovarian cancer samples and found that the gene expression of LRP1 enriched in tumor cells was highly associated with those of PSAP, A2M, CALR, and LARPAP1 in neighboring stroma cells, indicating the immunological importance of LRP1 and corresponding ligands. Efremova *et al*. introduced CellphoneDB, a general protocol for estimating cell–cell communication, together with a comprehensive ligand-receptor database [59]. CellphoneDB considered clusters of cells as different cell states and calculated the specific expression levels of receptors of a certain cell state versus ligands of other cell states in order to find meaningful ligand-receptor pairs. Later, Squidpy [60] implemented a faster version of CellphoneDB and utilized a more extensive ligand-receptor interaction database called Omnipath [61], extending the border for interaction annotation. Zhou *et al*. [62] applied CellphoneDB to the cancerous clusters and observed that the level of cell–cell interactions in metastatic tissues were more significant than in primary regions. Chan *et al*. compared the L–R interactions between small cell lung cancer (SCLC) subtypes using CellphoneDB [63]. They found 20 significant interactions in the subtype where NEUROD11 was differentially expressed (SCLC-N), which matched the morphological appearance that the cells were tightly adhered, whereas no significant interaction was found in subtype ASCL1 (SCLC-A). Cell–cell interaction analysis is a key step to understand the potential communications across different cell types. The ligand-receptor pairs found provide knowledge for drug usage, and new interactions can lead to the discovery of new predictive biomarkers and potentially new directions for drug development, which is crucial in forwarding precision medicine.

In summary, spatiotemporal omics is a powerful addition to the existing armory of multidisciplinary data integration for precision medicine. As ST technologies are continuously improving and a massive amount of data are being generated, algorithms tailored for ST are drastically advancing to deal with the computation, storage, visualization, and analysis challenges. Besides the sections above, cell fate and lineage are frequently explored in pathological studies. Trajectory and RNA velocity based on pseudotemporal ordering or mRNA splicing dynamics analysis can provide essential insights into cell fate decision processes. The application of trajectory analysis methods, such as Monocle [64], PAGA [65], Slingshot [66] aim to reconstruct the structure and dynamics of cell-stat transitions while RNA velocity methods, such as Velocyto [67], scVelo [68], Dynamo [69] predict the future expression status of cells. Recently, lineage tracer achieved through strategies which combine novel genome editing tools and computational methods is leading the more credible route of lineage segregation in studies on tumor progression [70], neural heterogeneity [71], stem cell biology [72], and so on [73]. With the development of spatial multiomics technologies, spatial multiomics integration algorithms will become the next hot spot. At present, spatial multiomics analysis are mainly focused on the integration of single-cell omics (scRNA-seq, snRNA-seq, ATAC-seq) and spatial transcriptome data. Most of the integration tasks aim to obtain the cell type distributions (for low-resolution ST data) or to obtain cell type annotation (for high-resolution ST data) of spatial barcoding capture spots. Existing methods introduced in section of Cell annotation are designed to reach that goal. With the emergence of spatial omics technologies, such as spatial proteomics technology and spatial chromatin accessibility technology, the integration algorithms of data from different omics will be required to be developed. Meanwhile, the combined mining of spatial multiomics which may focus on the regulatory mechanism exploration is also a big challenge. In the foreseeable future, integration of spatial multiomics analysis will provide an in-depth understanding of tissue structures, gene expression patterns, as well as cell–cell communication to aid diagnosis and therapeutic interventions, and complement precision medicine at the molecular level.

## The applications of spatiotemporal technologies in revealing the pathomechanism of disease

**Figure 3.**
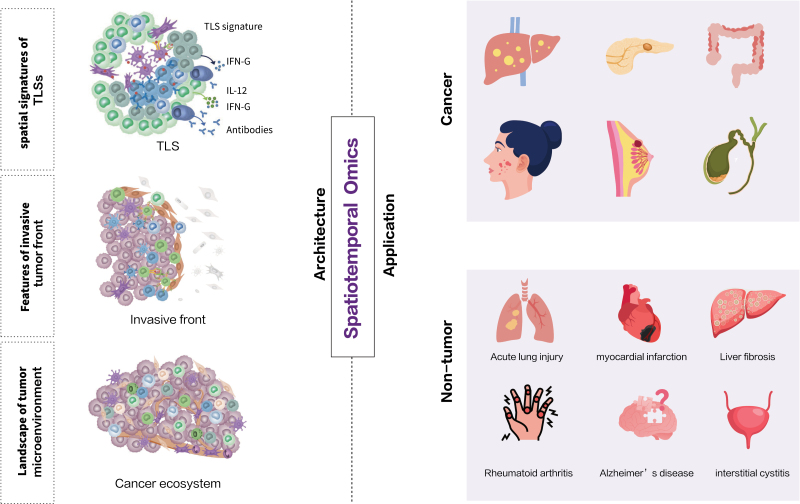
**Outstanding mechanistic investigations of diseases.** Researches in spatial context facilitate the understanding of underlying mechanism in different diseases by revealing molecular signature in featured architectures. Architecture depicted by spatiotemporal omics in diseases: (left) applications of spatiotemporal omics in dissecting cancerous pathologic structure; (right) scenarios of spatiotemporal omics in malignancy and nontumor disease.

Recent advances in omics technologies have inspired unprecedented efforts to characterize the molecular changes underlying the development and progression of a range of complex human diseases, especially in cancer. With precision medicine becoming the hot topic in disease management, oncology, and pathology are currently undergoing a wave of changes, including the need to assess an increasing number of biomarkers simultaneously to enable the recognition of more pronounced intratumoral heterogeneity. Moreover, it requires a complex dimensional mapping of biomarker expression within the TME. Spatiotemporal technologies have been widely used in discovering disease factors, establishing spatial maps, and drawing spatial blueprints. Currently, numerous studies on spatiotemporal techniques have provided a glimpse at the molecular organization of tissues and organs and how the disorder is associated with function and disease. This section will summarize representative studies which devote to resolving the spatiotemporal heterogeneity of tumor and nontumor diseases, providing new insights into the architecture and ecosystem in cancer, as well as spatial features of nontumor diseases (Table 2 and Fig. 3).

**Table 2.** Overview of key implications of spatiotemporal omics researches in precision medicine: assays, discovery, translation, and implication

Assays				Disease	Discovery	Publication	Translation and Implication
DNA	RNA	Protein	Morphology				
	√			Liver cancer	The distinct composition of TLSs was associated to the distance to tumor cells	Wu *et al*. Science Advances.2021	Describing composition and architecture of TLSs provided new insights in immune therapy.
	√			Breast cancer	Tumor-associated cell types were spatially mapped to find tertiary lymphoid-like structures. Colocalization of T-cells and macrophages was associated to type I interferon response.	Andersson *et al*. Nat Commun.2021	
	√	√	√	Renal cell cancer	Spatial transcriptomics revealed that anti-tumor antibody-producing plasma cells were produced and propagated in tertiary lymphoid structures.	Meylan *et al*. Immunity.2022	
	√	√		Squamous Cell Carcinoma	The authors identified a novel population of tumor-specific keratinocyte, status of immune infiltration and tumoral heterogeneity at leading edges.	Ji *et al*. Cell. 2020	Leading edge heterogeneity, cell states at invasive fronts and cellular crosstalk facilitated understanding of pathological alteration leading to metastasis and recurrence.
	√			Intrahepatic cholangiocarcinoma	The damaged states of hepatocytes indicated by overexpression of Serum Amyloid A (SAA) were identified at invasive fronts.	Wu *et al*. bioRxiv.2021	
	√			Liver cancer	The tumor capsule potentially affected intratumor spatial cluster continuity, transcriptome diversity, and immune cell infiltration.	Wu *et al*. Science Advances.2021	
	√			Pan-cancer	Recurrent tumor cell states spanning multiple cancer types were identified, which actively interacted with other cells in tumor microenvironment (TME).	Barkley *et al*. Nat Genet. 2022	Decipher Eco-system of cancer opened new opportunities for novel targets of treatment.
		√		Breast cancer	Spatial organization of the tumor-immune microenvironment within triple-negative breast tumors was linked to outcome of overall survival.	Keren et al. Cell. 2018.	
		√		Breast cancer	Ten TME structures were identified by vascular content, stromal quiescence versus activation, and leukocyte composition.	Danenberg *et al*. Nat Genet. 2022	
	√			Colorectal cancer	Spatially featured immune ecosystem uncovered the discontinuous inflammatory expression pattern in cancer regions.	Zhang *et al*. Fundamental Research 2022	
		√	√	Colorectal cancer	Cellular neighborhoods revealed spatial organization of the tumor microenvironment, and local enrichment of PD-1+/CD4+ T cells was positively correlated with survival in high-risk patients.	Schürch *et al*. Cell. 2020	
	√	√		Pancreatic ductal adenocarcinoma	TIGIT in exhausted and regulatory T cells and Nectin in tumor cells were coordinately expressed. Inflammatory cancer-associated fibroblasts that upregulated metallothioneins were enriched in chemo-resistant samples.	Zhou *et al*. Nat Genet. 2022	
	√			Alzheimer’s disease	Spatial transcriptomics identified a plaque-induced gene network and oligodendrocyte gene response in AD.	Chen *et al*. Cell. 2020	Decoding etiopathogenesis of nontumor diseases
		√	√	COVID-19	The disorganized structure of the infected and injured lung, alongside the distribution of extensive immune infiltration were identified in patients with COVID-19	Rendeiro *et al*. Nature. 2021	
	√			Myocardial infarction	A spatially comprehensive map of human cardiac tissue was characterized, and disease-specific cardiac cell states were profiled.	Kuppe *et al*. Nature. 2022	
	√		√	Liver fibrosis	Diseases-associated gene expression and distinct cell states within fibrotic regions were uncovered.	Chung *et al*. Hepatology Communications. 2022	
	√			Rheumatoid arthritis synovium	Comprehensive spatial RNA-seq data coupled to cell type-specific localization patterns at and around structures of infiltrating leukocyte cells in the synovium was reported.	Vickovic *et al*. Communications biology.2022	
	√			Chronic human tendon disease	Atlas of the dynamic cellular environment driven development of chronic human tendon disease was profiled by spatial transcriptome.	Akbar *et al*. Ann Rheum Dis.2021	
	√		√	Amyotrophic lateral sclerosis	Spatial transcriptome was used to identify disease-associated pathogenic mechanisms and establish the key steps in motor neuron degeneration observed in ALS.	Maniatis *et al*. Science. 2019	
	√		√	Breast cancer	The spatial transcriptome based signatures learned from expert selected breast cancer tissue sections by supervised machine learning methods can be used to classify breast cancer regions into distinguished carcinoma in situ and invasive ductal carcinoma.	Yoosuf *et al*. Breast Cancer Research. 2020	Spatial omics in diagnosis: Spatial omics provided a new vision in the search for key factors for the differential diagnosis to break the dilemma of clinic-pathogenesis lack in precision medicine.
	√	√		Pancreatic ductal adenocarcinoma	Interaction between FAP+ fibroblasts and SPP1+ macrophages regulated the remodeling of EMC, and consequentially resulted in pathological alteration of TME in CRC, which might be an indication of therapeutic efficiency.	Qi *et al*. Nat Commun. 2022	
		√		Non-small-cell lung cancer	This study demonstrated association of 12 spatially informed biomarkers to clinical outcome (PFS and/or OS) in NSCLC. Authors emphasized clinical potential of high CD56+ level in CD45+ compartment as the prognostic factor for PD-1 checkpoint blockade treatment.	Zugazagoitia *et al*. Clin Cancer Res,2021	Spatial omics in treatment decision: Decision of therapeutic regime required evaluation of markers from determined compartments or architectures by combing spatial, transcriptomic and proteomic information.
	√		√	Breast Cancer	Spatial RNA profiles could predict spatial intratumoral variabilities. A mRNA-based proliferation score showed an association with morphology and architecture with clinical potential.	Wang *et al*. Cancer Res. 2021	
√	√	√		Non-muscle-invasive bladder cancer	This study showed relevance of three omic signatures to risk prediction. It proved that higher immune cell infiltration was associated to lower recurrence rates with spatial proteomics analysis. The genomic and transcriptomic subtypes could independently prognosticate clinical risk.	Lindskrog *et al*. Nat Commun. 2021	Spatial omics in risk assessment: Spatial omics, as a promising solution, might efficiently prognosticate risk of metastasis and recurrence.

### Spatiotemporal technologies in the analysis of tumor spatial architecture

The spatial architecture of tertiary lymphoid structures (TLS) and invasive leading edges are of great value to decode the underlying mechanism of the invasion, metastasis, antitumor response, and prognosis [74]. Spatiotemporal technologies provide a new perspective for tumor research, allowing for comprehensive investigations of the spatial structure of tumors and seeking potential applications in clinical practice.

#### New insights in architecture: TLSs

TLS are identified in tissues of chronic inflammation and antigen retention, such as autoimmune disease, chronic infection, graft rejection, and cancer [75, 76]. In many cancer types, the presence and density of intratumoral TLS are associated with a favorable prognosis [74]. Resolving the spatial heterogeneity of TLS can help further explore the mechanisms underlying the tumor progression and antitumor response, which are insightful to assess the treatment response and clinical outcome.

ST has been used to profile TLS to provide new insights for understanding the function of immune cells in TLS. The integrated analysis of scRNA-seq and ST technology identified the spatially restricted type I interferon responses in the enrichment regions of T cell and macrophages in HER2-positive breast cancer [77]. And a predictive model based on the colocalization of B and T cells was constructed to infer the presence of TLS [77]. A study of renal cell cancer localized the architecture of TLSs with H&E staining, and further discovered the developing states from B cells to antibody-secreting plasma cells [78]. Then, the research reported that dissemination of plasma cells into tumor bed along fibroblasts track may induce tumor cell death, which contributed to the prediction of the therapeutic responses to immune checkpoint inhibitors and survival [78]. Rui Wu *et al*. [79] constructed a signature of 50 genes to identify TLS mainly existing in the leading edge area of primary liver cancer. Moreover, distinct composition of CD8^+^ Tcm and CD8^+^ Tem cell was significantly correlated with the distance from TLS to tumor cells [79]. Collectively, these studies provide the TLS signatures of different tumors at the ST level, and further investigate the function and status of immune cell subsets. It indicates that ST enables a precise analysis of specific structures within tumors, which will play an essential role in precise diagnosis in the future.

#### New insights in architecture: invasive leading edges

Invasive leading edges is recognized as the most active region for tumor cell infiltration and invasion, which helps to understand tumor invasion and metastasis. In the abovementioned liver cancer research [79], the researchers also investigated the microenvironment characteristics of tumor leading-edge sections, in which higher spatial continuity and lower transcriptome diversity were observed in tumor sides of samples with complete fibrous capsules. The authors observed that the integrity of capsules was closely related to the spatial heterogeneity of tumor cells, and to the distribution of surrounding stromal and immune cells [79]. In addition, Liang Wu *et al*. [80] observed the distinct cellular distribution, metabolic reprogramming of tumor cells, and inflammatory states of hepatocytes around invasive tumor fronts of intrahepatic cholangiocarcinoma patients. Spatiotemporal techniques also open up possibility of identifying new type of cells which contributes significantly to the function of tumor leading edges. A study of squamous cell carcinoma demonstrated that tumor-specific keratinocyte (TSK) populations were enriched at tumor leading edges, and that the immune infiltration and potential immunosuppression were observed at the heterogeneous tumor front by ST technology [81]. These studies profiled the heterogeneity, cell states, and cellular crosstalk at the invasive leading edge by spatiotemporal omics, which will contribute to the development of precision medicine by providing profound understanding of spatial feature of architecture and molecular signature on leading edges.

### Spatiotemporal technologies in the analysis of the cancer ecosystem

Traditional precision medicine approaches, such as whole-genome sequencing has difficulty in spatially distinguishing factors and cells in the TME within bulk tissues [82]. Although the development of single-cell sequencing technology has significantly enhanced our understanding of the TME, the spatial information remains a challenge. Recently, multiple studies have applied spatiotemporal omics technology to characterize the TME comprehensively. By analyzing the cellular composition, cellular neighborhood, and cellular crosstalk in the TME, a series of important discoveries have been obtained and significantly advanced the field.

In studies using single cell sequencing to investigate cell to cell communication, one unsolved problem is unproved authenticity of interaction due to the missing of spatial information. A pan-cancer study analyzed 15 types of cancer using the scRNA-seq data to define recurrent tumor cell states by gene expression modules spanning multiple cancer types, such as “stress” and “interferon response.” Further analysis of ST data suggested the defined cancer cell states actively interacting with multiple cell types such as immune cells and stromal cells in TME [83]. One MIBI-TOF study of breast cancer provided the TME landscapes of structured tumor-immune border and spatial alternation in tumor progression [20]. The key findings are critical interactions between tumor and immune cells and profiles of immunoregulatory proteins in distinct cellular subtypes, which may provide evidence for the precise classification in breast cancer [20]. The spatiotemporal omic technique can not only use information like architecture, but also allow spatial analysis at cellular level. Esther Danenberg *et al*. [84] highlighted the complex TME structures and revealed that the co-occurrence of Tregs and dysfunctional T cells was linked to the inferior outcomes of higher hazard ratios for ER-positive breast cancer survival [84]. Daniel Cui Zhou *et al*. [85] identified the expansion of inflammatory cancer-associated fibroblasts in chemo-resistant patients and observed the immunosuppressive TME of Pancreatic Ductal Adenocarcinoma (PDAC). Taken together, these pioneering spatial transcriptome studies in various complex diseases fully demonstrate the potential of ST in disease research and diagnosis, and will encourage researchers to conduct more ST in disease studies.

### Spatiotemporal technologies in the analysis of nontumor diseases

Not only in the field of precision medicine for tumors, but some nontumor diseases also have difficulties in dissecting the complex mechanism, such as limited-sized samples with complicated pathological conditions. Here in current section, we summarize representative researches using the ST technology in revealing the mechanism of nontumor diseases.

Taken Alzheimer’s disease (AD) as example, the spatiotemporal techniques could offer a unique method of unraveling the dysregulated cellular network associated with AD and other brain disorders. Bart De Strooper *et al*. [86] investigated the transcriptional changes occurring in tissue domains in a 100-mm diameter around amyloid plaques in mouse model. The result showed coordinated cellular response around amyloid plaques, which was then confirmed by ISS on mouse and human brain sections [86]. Therefore, this study provided great value to subsequent studies of brain disorders. Molecular alteration in pathological region is critical in decoding the etiopathological reason of disease. A team of researchers investigated the cellular composition and spatial architecture of acute lung injury (including SARS-CoV-2 infection) in humans [87]. These spatially resolved data unveiled the disordered structure of an infected and injured lung, as well as extensive immune infiltration [87]. Christoph Kuppe *et al*. [88] provided an integrative molecular map of human myocardial infarction and observed the myeloid cells expanding in ischemic regions with higher NFκB signaling activity. In the study of in human liver fibrosis [89], diseases-associated distinct gene expressions and distinct cell states within fibrotic regions were uncovered by ST technology. Moreover, Liao Peng *et al*. [90] revealed the activation network of immune cells in interstitial cystitis (IC) bladders, then ST analysis localized these immune cells in the urothelial region or close to fibroblasts in IC bladders. With advancing of technique, the spatiotemporal omic technique extends its application in researches of muscularskeletal diseases. Sanja Vickovic *et al*. [91] studied interactions at the site of chronic synovial inflammation in rheumatoid arthritis patients. Moeed Akbar *et al*. [92] analyzed cell–cell interactions to reveal the dynamic cellular environment that drives chronic human tendon disease by using scRNA-seq and ST. The study suggested that the variations of immune cells and stromal compartments may cause dysfunctional immune homeostasis, which leaded to the development of chronic human tendon disease [92].

In general, the utilizations of spatiotemporal omics in mechanistic investigation shows the outstanding capacity to expand the knowledgement of cellular and molecular alternation in diseases, which greatly encourage both researchers and clinicians to develop the precision medicine for better diagnosis, treatment, and management strategies.

## Spatiotemporal omics: time to facilitate precision medicine

**Figure 4.**
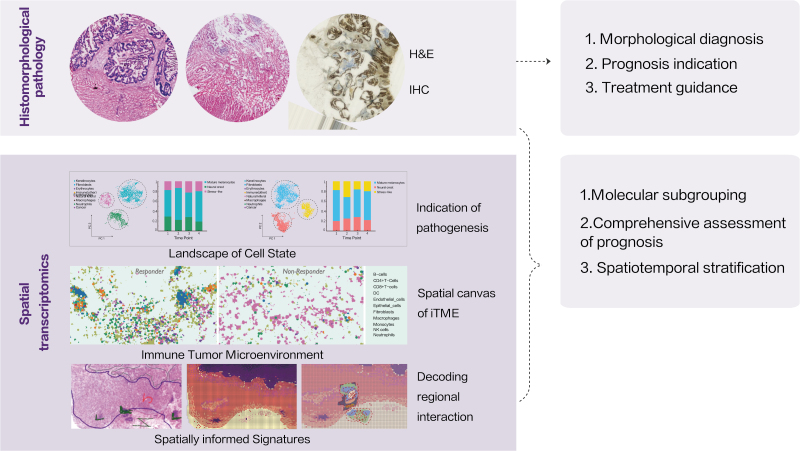
**Spatial signature: from bench to bedside.** Comparing to limited knowledge provided by histomorphological pathology, spatial signature possesses great potential of translation into clinical application: (up) morphological anatomy of tested tissue by traditional pathological examination; (down) identification of distinct cell states in malignant pathogenesis; compartmentalized iTME between immunotherapy responder and non-responder; decoding intercellular crosstalk in tumor edge.

Complex diseases comparable to cancer complicate the streamline of precise disease management, including diagnosis, stratification, treatment decision, and prognosis. This is due to the predisposition of the changeable genome and the interaction between genetic and environmental factors. The highly dynamic nature and multifactorial pathomechanism make clinical diagnosis and treatment decisions hardly manageable. Spatiotemporal omics technologies have profound implications, not only for differential diagnosis and treatment guidelines but also for risk assessment and pathogenic mechanisms research. Conclusively, spatiotemporal omics technologies provide impetus to improve precision medicine (summarized in Table 2 and Fig. 4).

### Spatiotemporal omics promises a pathogenesis-related diagnosis

As discussed in section 4.3, spatial information contributes significantly to revealing the origin and development of tumor cells. Therefore, the emergence of spatiotemporal omics can bridge the gap between tissue architecture manifested by disease and underlying molecular profiles as a promising diagnostic or early detection modality for precision medicine [93]. Here, this section summarizes representative researches in clinic-related setting to create a canvas of biomolecules which may potentially translate into early diagnosis of cancer and restratification of disease.

#### Spatiotemporal omics discovers new spatial signature for early detection of cancer

Early detection of cancer has been well acknowledged for its essential role in public health to improve wealthfare and reduce economic burden. However, poor accuracy, low efficiency, unsatisfied specificity, and instable consistency hinder the application and accessibility [94]. Plasticity of cell state has been proved to manifest during transcriptomic and genetic evolution [95]. With ST, Baron *et al*. [95] recently identified stress-like cell subpopulation with expression of fos and jun, which balanced between cell survival/quiescence and apoptosis. Therefore, this study demonstrated fos and jun expressed in newly defined stress-like population as signatures for melanoma tumorigenesis at early stages [95]. With the help of scRNA-seq and ST, Ji *et al*. [81] constructed a transcriptomic atlas of cutaneous squamous cell carcinoma (cSCC) subpopulations and integrated spatial profiling to assess the cell niches. From their findings, they indicated a TSK subpopulation with widespread tumor-specific gene inducement exclusive in cSCC to distinctly differ from normal counterparts, which, therefore, highlighted the potential of TSK in cancer candidate [81]. Moreover, Wang *et al*. [96] uncovered novel mechanisms underlying gliomagenesis by utilizing Hi-Chip and ATAC-seq to spatially interrogate tumor structural and epigenetic landscape. Their identification of potential enhancer hijacking and gene co-amplification, including FLRT1, A2M, and JAG2, implied the significance of three-dimensional genome alterations in pediatric high-grade gliomas’ epigenetic landscape and tumorigenesis [96]. The assay of spatiotemporal omics provides new insights to understand the cell type-specific biomarkers, which are associated with tumorgenesis dynamically and multiomically. The screened cell type or spatially specific targets need independent cohort to validate the precision, sensitivity, and accuracy. Therefore, spatiotemporal omics is capable to develop new classes of spatial biomarkers rapidly and accurately under study design with clinical question driven.

#### Spatiotemporal omics facilitates precisive stratification of diseases

Spatiotemporal molecular medicine has shown a wide range of application scenarios in neoplasm and non-neoplasm diseases, including rare diseases, incurable neuro-degenerative, and developmental diseases [97, 98]. Tumors like breast cancer with resembling histological appearance can be of different causes and responses to treatment [99]. In a recent study, supervised machine learning methods were leveraged to classify breast cancer regions into distinguished carcinoma in situ and invasive ductal carcinoma based on their spatial transcriptome profiles [98]. It indicates the importance of including molecular profiling to lead to a spectrum of subtypes with biological relevance. Another research on the rare disease of amyotrophic lateral sclerosis (ALS) applied ST to obtain gene expression of mouse spinal cords and postmortem tissue from ALS patients, which described the potential pathogenic mechanisms and offered novel clues for ALS in the differential diagnosis and treatment [97]. The stratification of rare diseases is a clinic problem due to limited access to patients, spatiotemporal omics can feature molecular diversities and subclasses of cell types, breaking the border of precisive stratification. Conclusively, spatiotemporal omics has provided a new dimension containing spatial and molecular information in searching for key factors and associated panels that function significantly at the pathogenic mechanisms. It could serve as a novel biomarker system for differential diagnosis to breaking the dilemma of clinic-pathogenesis lack in precision medicine [100].

### Spatiotemporal omics rewrites the modalities of disease intervention

Sequencing sets a milestone in clinical diagnosis and represents the new era of treatment regime by shifting from manifested symptoms to underlying genomic changes [101]. Increased data accrual and decreased expense of sequencing streamline precision medicine in different aspects of early screening, diagnosis, treatment, and prognosis. With molecular diagnosis distinguishing patients eligible for targeted therapies, the therapeutic benefit remains within a minor population, which causes a great economic burden [102]. From 2006 to 2020, although patients’ eligibility for genome-targeted therapy increased from 5.13% to 13.60%, the response of patients increased only from 2.73% to 7.04% [103]. It is speculated that resistance to treatment and uncontrolled toxicity contribute to this unsatisfactory clinic consequence [102, 104].

Low objective response rates and acquired resistance to treatment have become key limitations in immunotherapy treatments. One of the major reasons is the lack of effective predictors for companion diagnostic [105, 106]. A recent study revealed a transcriptomic landscape of both immune and nonimmune cells in the microenvironment of colorectal cancer using single-cell RNA sequencing and ST [107]. Coincidentally, Zugazagoitia *et al*. [108] also reported a number of relevant immune predictors in the spatial context identified by the digital spatial profiling technology (GeoMx). Their results demonstrated the potential of using spatially informed biomarkers to predict response to PD-1 checkpoint blockade in non–small cell lung cancer (NSCLC) [108]. Patient stratification in immunotherapy has been relying on a single or a small panel of biomarkers, which is evidently not precise enough to predict the immune response and efficacy of immune checkpoint blockade in various tumors [109]. A growing body of evidence suggests that future companion diagnostic tests for predicting immunotherapy efficacy require evaluating markers from particular tissue compartments or architecture by combining spatial, transcriptomic, and proteomic information [100, 108].

### Spatiotemporal omics in risk assessment: metastasis and recurrence

Metastasis and recurrence are the leading causes of mortality in cancer patients. With increased understanding of the underlying mechanisms, the abilities of tumor cells to infiltrate, invade, and destruct adjacent tissue are believed to be associated with the risk of recurrence and metastasis [110]. As discussed in section 4.1, biological behaviors of cells at invasive leading regions predominantly decide the tumor’s fate as cells remain at the origin site or migrate to distant sites. Taking the colorectal carcinoma as an example, intra- and peri-tumoral budding are significantly associated with local recurrence, lymph node, or distant metastasis [111]. The identification of budding and prognosis of recurrence and metastasis rely on histomorphology assays with limited traces [111]. A study with CODEX used spatial analysis of cellular neighborhood to suggest the expansion of PD-1^+^/CD4^+^ T cells in the cellular neighborhoods of granulocyte enrichment were positively correlated with improvements in survival outcomes [112]. In addition, this section will summarize novel insights to facilitate tumor prognosis of the results of spatiotemporal omic studies.

With the support of spatiotemporal omics, in situ and high-resolution analysis of all mRNA in a single tissue section can be realized. Obtaining the expression, localization, and differentiation of these functional genes in specific tissue regions enables researchers to decode the interaction among tumor cells, immune cells, and stromal cells. Together, these help to find risk factors for metastasis, recurrence, and poor prognosis. A recent study by Jamieson *et al*. [113] unveiled the immune landscape of PDAC utilizing the DSP. This study proved the potential to identify spatially informed biomarkers with prognostic relevance in biopsy tissue-sized samples from treatment-naive PDAC [113]. Combined analysis of ST and proteomic approaches exhibited clear superiority in the clinical research of TMEs. Another study of breast cancer predicted spatial intratumor variabilities in gene expression and validated the findings through ST profiling, demonstrating the robust prediction ability on proliferation score and potential clinical value [114]. As is well known, proliferative signatures such as Ki-67 mitotic index and proliferating cell nuclear antigen (PCNA) have been used to assess proliferation and appear as prognostic factors in traditional pathology [115, 116]. It is believed that proliferating signatures are one of the most reliable predictors of prognosis in the development of the outcome predictor score [117]. Here, spatiotemporal omics offers a novel comprehensive approach to evaluating proliferative and prognostic risk assessment.

Diagnosis and patient stratification remain critical clinical challenges in precision medicine. We believe that spatiotemporal omics combining transcriptomics, proteomics, morphology, and potential epigenetics is a promising solution with detailed information on gene expression and location to efficiently improve clinical outcomes.

## Challenge and landscape of spatiotemporal omics in precision medicine

The concept of precision medicine was proposed in 2015 first time. Nowadays, growing knowledge of human genetics is changing how physicians and clinicians predict cancer risk and determine treatment regimes. Along with genomic sequencing, diagnostic tests such as FISH, PCR, and systems biology, combined with machine learning algorithms and digital imaging, assist clinical diagnosis at the molecular level. It results in the possibility of precise medical products with determinant targets to achieve optimal treatment. However, the treatment response and efficiency might not meet the expected outcome. These technical limitations impede the identification of cells responsive to early cancer cell colonization and the development of good therapeutic strategies.

In the current review, the authors proposed the clinical implication of the state-of-the-art omics analyses, which have been proven to uncover key mechanisms of cancer development, treatment resistance, and risk of recurrence. The challenges of translating spatial omics from bench to bedside are evident. Massive quantities of data need an empowered algorithm to mine useful and clinicopathologic-associated information to make the data accessible and readable. As reviewed in section 5, multiomic information has the potential to elaborate biological and functional status, reflecting the TME landscape. A mature toolchain is desired to visualize data into a quantitative dataset, identify essential cell phenotypes, and map spatial and cellular neighborhoods.

Spatiotemporal omics has been proved to have a unique role in exploring the molecular mechanisms of embryonic development and differentiation, organogenesis, and pathological change in the tissue, enabling real-time monitoring of dynamic changes in gene/protein expression and intercellular and inter-tissue communication [118], in which the information of time has been used. Limitations in dissecting omics with temporal dimension in clinical translation is due to the lack of accessible acquisition for clinical samples at proceeding timeline. Temporal information has been widely explored in studies of development to dissect dynamic signature of cell lineage. Other than sampling at different time points, certain algorithm has been developed to deduce temporal analysis like lineage tracer. Lineage tracer provided information of tumorigenesis by recounting the history and chronology of critical events during cancer progression. However, it has been so far scRNA-seq based analysis. The application of lineage tracer like algorithm in spatiotemporal omic data is a hot topic and eager to be resolved. Further development in algorithm will promisingly ensure spatiotemporal to illustrate at different dimension.

**Figure 5.**
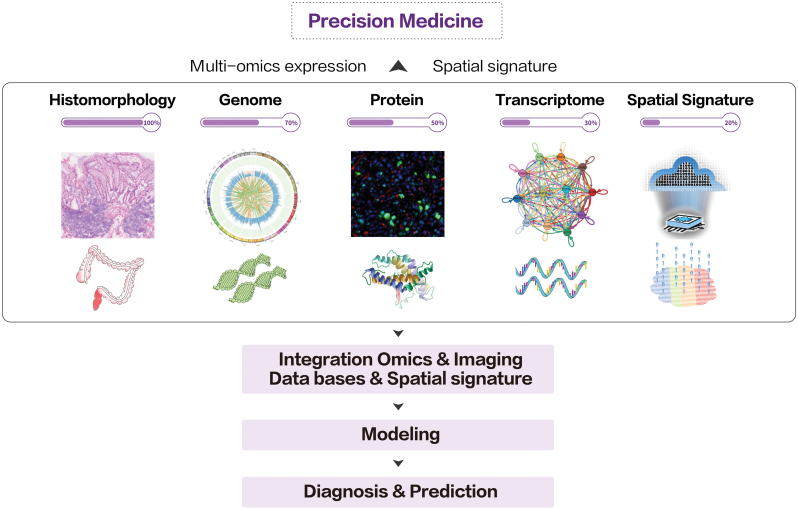
**Schematic diagram of spatial precision medicine.** Landscape of omics data contributes to the practice of precision medicine in the future. Integrated analysis of integrated multiomics will promise a new modalities of clinical practice in precision medicine. Each technique for omic detection need to be proved its accessibility for clinic. The progression has been indicated by the estimated percentage of completion in this figure.

Researches with spatiotemporal omics have provided novel insights into the pathomechanism of tumors by analyzing the spatial architecture, mapping the complex organization, and decoding essential interactions, mainly in research settings to date. Present publications promise a reasonable expectation of improvement brought by spatiotemporal omics in the effectiveness of therapies and prevention strategies. Genuine concerns are the accessibility due to expense and the reproducibility due to complicated procedures. Companies like Akoya and 10× provide solutions with automatic detection of analytes, which eliminate noises caused by batch effects. The end-to-end solution can not only guarantee batch-to-batch standardization but also reduce the manpower cost, turning spatial omic technologies into actionable intelligence (summarized in Fig. 5).

Clinical sequencing technologies have been seen as explosive growth. The technologies are capable of collecting morphological and spatial information together with omic data. With novel research insights, it will streamline the R&D of new drugs, the discovery of biomarkers, and finally, the design of clinical trials. An end-to-end solution of spatiotemporal omics with satisfactory reproducibility, sensitivity, throughput, and price will assist physicians in making accurate diagnoses, determining an effective treatment, avoiding wasting time, and minimizing toxic impact. Breakthroughs in precision medicine can be expected to improve care delivery, health outcomes, and quality of life. The future of precision medicine is not here yet for spatiotemporal omics-based companion diagnostics, but it is coming.
